# Reaching out to fathers in Afro-Caribbean contexts: a case study review of best practices from the Fatherhood is Sacred program in native communities

**DOI:** 10.3389/fpsyg.2024.1363173

**Published:** 2024-07-24

**Authors:** Suzette Hudson, Sean E. Brotherson

**Affiliations:** Department of Human Development and Family Science, North Dakota State University, Fargo, ND, United States

**Keywords:** fathering, native fathers, historical trauma, Afro-Caribbean families, Fatherhood is Sacred

## Abstract

Historical trauma has played a significant role in the difficulties of fathers to fulfill their coparenting roles in Native American communities. This pattern is also true for men in Afro-Caribbean communities. Fatherhood programs developed by the Native American Fatherhood and Family Association (NAFFA) have shown effectiveness in supporting fathers, enhancing their confidence and coparenting skills, and overcoming trauma in Native communities. This paper seeks to identify the opportunities and best practices for cross-cultural adaptation of the Fatherhood is Sacred program to Afro-Caribbean families and contexts.

## 1 Introduction

Systemic adversities have troubled Native American families and communities for generations. The available literature recounts the persistent loss of lives, land, language and cultural identity among these populations. Native American individuals, families and communities have experienced repeated episodes of ethnic and cultural disruption, conflict and even genocide (Evans-Campbell, 2008). The results have included lasting disruptions in the indigenous family systems which focused on the active engagement of both parents in raising children (co-parenting), alongside other family members.

The literature on family systems theory gives special importance to the coparenting relationship. Parents are seen as the family’s executive system. The effective functioning of this system provides children with a sense of predictability, stability, and security in the family (Holmes et al., 2013). Research on the impact of coparenting is relatively new, but initial indications are that coparenting contributes to the well-being of the child over and above the sole effects of maternal or paternal parenting (Boričević Maršanić and Kušmić, 2013). In this paper, we will highlight the effects of two coparenting models within distressed Native American and Afro-Caribbean communities. This section provides background and context on the family and cultural settings in which a potential intervention might be implemented. Then, we will reflect on and distill critical lessons and best practice possibilities that can be learned from the Fatherhood is Sacred program (Native American), which encourages the re-engagement of paternal parenting as a critical element to the coparenting alliance. Implications for application of such lessons and practices for use with men and families in the Afro-Caribbean community will be discussed.

## 2 Context

### 2.1 Family system shifts, single parenting and fathering in native communities

From the mid-19th century through to the 1970s in the United States, federal policy included the creation of boarding schools to support the forced assimilation of Native Americans (Zephier Olson and Dombrowski, 2020). Native youths were forcibly taken from their families from as early as 5 years of age, with reports of exposure to child abuse, while spiritual and cultural practices were prohibited among Native peoples. Diseases resulted in widespread death, while government policies led to the establishment of under-resourced reservations (Thornton, 2005). Scholars have reported that such historical pressures “had a profound and traumatic impact on the Native American family unit and the tribal community” (Shears et al., 2011, p. 203).

This bleak narrative has continued into the modern day, with varied Native communities recording some of the highest rates of poverty, violence, substance abuse, incarceration, and parental absence in the United States (U.S.) (Centers for Disease Control and Prevention, 2019; Richards et al., 2021). More recently, U.S. Census data has revealed the prevalence of single-parent families in Native families, headed primarily by the matriarch. On some reservations this phenomenon has become a growing trend that has persisted over time (Sandefur and Liebler, 1996). A recent demographic analysis by the U.S. Administration for Children and Families of 2015 American Community Survey (ACS) data notes: “Almost 41% of Native American children in tribal areas with a child support program lived in female-headed single-parent families. Another 12% lived in male-headed single-parent families. Combining these two-family types, we find that 53% of Native American children in tribal areas with a child support program lived in single-parent families. In contrast, only a third of all American children lived in single-parent families: 26% in female-headed and 7% in male-headed single-parent families” (Sorensen, 2023). Often without the benefit of co-parenting relationships in these family settings, Native American children in single-parent families experienced twice the rate of poverty (54%) than their peers in married-couple families (22%) (Sorensen, 2023).

Juxtapose this contemporary reality with traditional Native American cultures where families were seen as being sacred. Strong families were the foundation upon which Native Americans transmitted their traditions, heritage, and culture. Families included a wide circle of relatives linked together in kinship and mutual dependence, going beyond the nuclear family to include grandparents, uncles, aunts, cousins and many others, forming an extended family unit (Pritzker, 2000). By extension, traditional tribal communities also viewed children as sacred beings, placed at the center of the Nation, and were to be protected within the tribal community. Fathers were involved in providing for families (though “breadwinning is a concept that emerged with industrialization, urbanization, and the separation of the home from the workplace) (Pfau-Effinger, 2004), but they also served as figures of authority, providing guidance, spiritual leadership and the transmission of communal rituals, strong values and life skills to children (Brave Heart et al., 2012; Deer, 2015). Among the Lakota peoples, for example, fathers and father figures acted in the role of *wicasa was’aka* (“strong men” in Lakota), or as the warriors and protectors of their children, families, and nations (Brave Heart et al., 2012). The term *historical trauma* has been employed to explain the significant and long-lasting exposure to displacement, violence and adversity that has been visited upon Native peoples for centuries (Brave Heart et al., 2012).

Our understanding of the full direct and indirect effects of traumatic events of this nature on individuals, families and communities is still limited. However, it is clear that the healthy functioning of families with the active involvement of men as fathers, and the social structures to support thriving families and communities, have been disrupted within Native American communities (Pritzker, 2000). The intergenerational effects of historical trauma have given rise to family patterns that undermine coparenting and extended family support in Native communities, putting many American Indian children at risk. Extensive research has shown that the absence of a father from the home is associated with developmental challenges in children, including developmental delays, teenage pregnancy, delinquency and physical as well as emotional abuse (McLanahan, 2014; Chavda and Nisarga, 2023). Also, father absence or under-involvement is strongly associated with risk of juvenile justice difficulties for youth (Major et al., 2004; Rolnick, 2016).

We would suggest that any attempt to make sense of the challenges facing Native American families, fathers and communities, as well as any effort to respond to these challenges, must consider the possible direct connection to past traumatic events (historical trauma), the transmission of these events through generations and the compounding of this situation by persistent contemporary traumatic events.

### 2.2 Can historical trauma explain shifts in co-parenting norms in ethnic communities?

*Historical trauma* has been defined as a kind of “collective complex trauma” which is imposed on a group of people who share a common identity or affiliation, e.g., ethnicity, nationality, or religious affiliation (Evans-Campbell, 2008). The members of the affected community share a similar experience, usually loss, with the majority displaying a similar set of reactions linked to trauma. The resulting legacy is usually that of psychological and social responses to traumatic events. There is a substantial body of research in the developmental science field that points to epigenetic processes mediating the intergenerational transmission of trauma effects, resulting in the biological embedding of adverse experiences across generations (Yehuda and Lehrner, 2018; Švorcová, 2023). Continuing research is likely to further illuminate how such impacts that persist at the genetic level across time may be significant in adaptive reactions to stress within communities such as Native America or the African diaspora.

In Native communities, it is suggested that despite tremendous resilience being displayed, the effects of historical trauma “have had a toll, not only on individual mental health but also on the healthy functioning of families and AIAN (e.g., American Indian-Alaska Native [AIAN]) social structures as a whole” (Evans-Campbell, 2008, p. 317). Much of the initial scholarly work related to historical trauma dates back to studies on Jewish Holocaust survivors and their descendants – the “survivor syndrome” and the survivor child complex (Brodaty et al., 2004).

The work of Brave Heart and Chase (2016), as well as other scholars, presents evidence to suggest that historical trauma has affected Lakota parents and children by changing parenting behavior, with the adverse effects of placing children at risk for alcohol and other substance abuse. For example, research findings reveal that Lakota Indian parents who were raised in boarding schools felt a high level of inadequacy as parents, as a direct effect of the trauma experience, and in turn were often unable to provide the support needed by their own children. Their children in turn reported a history of neglect and abuse from parents who were unfamiliar with and unable to demonstrate healthy parenting styles, in contrast to healthy coparenting and extended family models that previously existed among the Lakota. The normalization of impaired parenting behaviors (which included non-nurturant and ineffective parenting and poor bonding with family, as well as uninvolved parenting), resulted in the perpetuation of risk behaviors among youth through multiple generations (Brave Heart, 1999; Brave Heart et al., 2012; Brave Heart and Chase, 2016). This behavior has been reinforced over the years by being transmitted in indigenous communities where traditional parenting values and culture have been eroded. The perpetuation of similar trauma effects in varied Native communities has led to strained and altered social systems that often result in absentee fathering and limits healthy coparenting in these Native American communities. Unfortunately, a similar tale has been played out and now persists in Afro-Caribbean communities.

### 2.3 Historical trauma and Afro-Caribbean family systems

In the Caribbean, slavery, colonization, indentured servitude and racism have marked the history of Afro Caribbean peoples from as far back as the 1500s. Enslaved people could not legally marry as, by colonial laws, they were considered property and not legal people who could enter contracts such as marriage. Some families were non-nuclear, with fathers owned by one plantation master and the children he fathered, as well as the children’s mother, owned by another plantation master, miles away. In these instances, family bonding and father involvement were not encouraged (Klein and Vinson, 2007; Morgan, 2016). Enslaved people lived with the perpetual possibility of separation through the sale of one or more family members. Slaveowners purposefully separated children from their parents to blunt the development of affection between them (Bush, 2010). The legacy of enslavement of African peoples in the Caribbean persisted beyond its abolition, with economic hardship for Afro-Caribbean peoples resulting in “a system of migratory labor between islands that took men away from their homes” (Bush, 2010, p. 86). This led to the common stereotype of the “mother-headed, black family characterized by the absent, irresponsible black man and negligent parenting” (Bush, 2010, p. 86). For families seeking to manage their problems and survive through a dependence on kin networks and group support that persisted despite a history of trauma, these lasting effects of displacement and degradation simply continued to strain family patterns and re-fashion them into unhealthy models.

The result of this lasting trauma has been an Afro-Caribbean culture which is replete with patterns of matriarchal single-parent households, common-law marriages, visiting fathers and child-shifting (where other persons besides the parents are given the responsibility of taking care of children). Roopnarine (2013) notes that non-married mating unions in the Caribbean were common, with marriage rates in three different samples in Jamaica being ranging between 23.7 percent and 37.3 percent. The concept of coparenting does not require marriage as a precursor to responsible parenting (McHale et al., 2004). Instead, there must exist a high degree of coordination and support between adults rearing a child together. In the Caribbean, among Afro-Caribbean populations in Jamaica, it is estimated that more than 47 percent of children live in single-parent homes with their biological mothers (Headley, 2021). This cultural context appears to have emerged as a result of family shifts due to slavery and other traumas, and thus provided the basis for absentee fatherhood in Afro-Caribbean communities with this social phenomenon being normalized by the presence of modern-day traumatic experiences including poverty, racism and persistent unemployment (Jemison, 2015). Of note, research into mediating factors such as extended family support systems or social fathers that serve to mitigate the impact of absentee fathers on children in these communities is limited.

Degruy-Leary (2017), writing reflectively about a similar family pattern of absent fatherhood shared by many African Americans in the United States, posits the concept of *post-traumatic slave syndrome* (PTSS). According to Degruy-Leary (2017), PTSS is a variation of historical trauma that describes the multigenerational trauma and injustices unfairly visited upon African Americans. The emotional and psychological effects include, among other difficulties, the experience of displacement and troubled relationships between fathers and their children. Although there is some critique of this perspective, its relevance here relates to the linkage with other frameworks of historical trauma and their application to specific groups, in this case those of African descent whose ancestors endured slavery. Degruy-Leary (2017) further argues that post-traumatic slave syndrome has been passed down through generations and continues to affect many individuals and families in the African diaspora today, which would include those in the Caribbean. The forced separation of fathers from their families during this painful period in history established a pattern that continues to be evidenced today in the tendency among Afro-Caribbean fathers to be separated from their families (Jemison, 2015).

Recent government data in 2019 from the Planning Institute of Jamaica noted that “an estimated 36.4 per cent of children had no father figure in the home compared to 2.1 per cent with no mother figure” [The Planning Institute of Jamaica (PIOJ) and The Statistical Institute of Jamaica (STATIN), 2022, p. 2], leaving many Caribbean children without a meaningful father presence in their lives.

From a developmental science perspective, a key question focuses on how we can develop systems and intervention programs to treat and heal individuals and families affected by historical trauma. Undoubtedly, any solution must be community based, given the far-reaching effects of the trauma, and must encompass culturally informed solutions to repair the damage done to the loss in identity, familial stability and cultural cohesion. In the cases noted here, particular injury has accumulated in the roles and relationships of men as responsible fathers in their families, and so information that offers best practices to follow in mitigating negative effects and improving resilience has promise for application in these communities.

## 3 An overview of the Fatherhood is Sacred program

In 2002, Albert Pooley, President of the Native American Fatherhood and Families Association (NAFFA), founded the Fatherhood Is Sacred^®^ program to address the challenge of father absence and under-involvement within Native American communities. Pooley noted that government policies and programming efforts in the United States were primarily “focused on the well-being of Native American women and children, with little attention being paid to Native American men” (Montgomery, 2015, p. 91). To focus more resources on assisting men in their development and strengthening their family systems, Pooley developed an intervention program that sought to promote the importance of responsible parenting among Native American families and communities. More specifically, the program focused on healthy fathering and bringing back the building blocks of culture, heritage, tradition, and Native spirituality. The overall purpose of the programs supported by NAFFA are to “re-establish the close familial ties of Native Americans, including the role of Native American men as leaders within their family and community” (Montgomery, 2015, p. 92).

The Fatherhood is Sacred program identifies the need for healthy men in Native American families and communities today. To facilitate this goal, it embraces the importance of healthy parenting and generative fathering, with the main objective of the program being that of “reinvigorating and uplifting the male population’s role within the family setting” (Brotherson et al., 2005; Montgomery, 2015, p. 91), contextualizing their role as fathers against a renewed focus on culture and spirituality. Specifically, the program seeks to empower, train and support Native American men and fathers so they understand that: (1) families are sacred and should be treated as such; (2) they are not the problem in their communities, but instead, are the solution; (3) they possess innate leadership abilities and their cultures intrinsically value families; and (4) their role, involvement and leadership as fathers is critical to keeping families together and ensuring their wellbeing (Montgomery, 2015; Pooley, 2021). In this regard, the program has three core pillars: self-worth, identity, and purpose. These pillars are embedded throughout the 12 individual sessions of the curriculum. Building a strong sense of self-worth, cultural identity, and life purpose as a father is central to the program. The program also encourages relationship building, with activities geared towards improving family bonds and responsible parenting, with a curriculum that is delivered through a series of twelve group sessions that allow for discussions, critical thinking, and problem-solving around fatherhood and family issues. Through this programming, traditional values are restored, a sense of identity, direction, purpose and self-worth is rekindled in men, and they come to view their role as critical to the development of their families and by extension, their communities (Pooley, 2021). For more specific information on the program, details and curriculum materials are available through the Native American Fathers and Families Association (see https://www.nativeamericanfathers.org/).

The intervention programs supported by NAFFA explicitly acknowledge the impact of historical trauma on family systems in Native communities. For example, Goodrow (2015) notes in an evaluation of the program that family roles in Native communities “have been greatly impacted by the history of emotional and psychological injury and genocide of Indigenous cultures over the generations,” and further states that such impacts have been “especially significant to the development of the sacred roles of mothers and fathers, the feeling of cultural belonging, and the loss of traditional practices” (p. 1). As a response to these historical impacts, the Fatherhood is Sacred approach encourages participants to “stand on their own goodness” (Goodrow, 2015, p. 4), which means they must recognize and affirm the character, resiliency and cultural heritage that have enabled their generations to survive and affirm the centrality of family. The program’s intent seeks to provide hope, inspire gratitude and increase understanding among the men as a means of bringing about change in behavior and attitude and encouraging self-motivation, notwithstanding the current context of their daily lives (Montgomery, 2015). Program facilitators lead sessions on topics such as nurturing the entire family, linking generations, strengthening individual and cultural identity, and the benefits of service to family and community (Montgomery, 2015; Pooley, 2021). The Fatherhood is Sacred Program has been used extensively by tribal communities in the United States, Canada and beyond, and has been recommended by government entities and agencies for its “best practices” in encouraging responsible and involved fatherhood (Goodrow, 2015; Montgomery, 2015; Sarche et al., 2020).

The adoption of the Fatherhood is Sacred program has been widespread in the United States. For example, Project LAUNCH (Linking Action for Unmet Need in Children’s Health) furnishes federal grants to support child health and wellness to a variety of grantees, and particularly “facilitate increased access and use of evidence-based prevention and promotion practices” (Sarche et al., 2020, p. 1). For tribal LAUNCH grantees serving Native American populations, a key priority has been to facilitate culturally grounded and relevant approaches to serving children and families. By 2020, 40% of grantees reported using the NAFFA programs in their efforts, which was the second highest usage among 18 different models or intervention programs documented (Sarche et al., 2020). Another brief summary about the program indicated that by 2015, over 300 facilitators had been trained and “implemented the program in over ninety tribes and twelve urban centers across the nation” (Montgomery, 2015, p. 92).

Reviews of the program have noted its success in helping with recovery from addiction, increasing understanding of historical trauma, facilitating healing and enhanced spirituality, increasing connection, and supporting healthy coparenting relationships within American Indian and Alaska Native families (White and Brotherson, 2005; Goodrow, 2015; Wilson et al., 2022). Best practice ideas and implications from this program are explored in the sections that follow.

An initial quantitative evaluation of the Fatherhood is Sacred program, conducted by White and Brotherson (2005), surveyed program participants at two sites in the southwestern United States. Respondents included 84 Native American men who participated in the program. Nearly 80% of these men reported challenges with substance misuse and broken relationships with their children (White and Brotherson, 2005).

While most men surveyed about the program indicated its positive value to them and 94% would recommend the program to other men, also 93% indicated that they would benefit from additional supportive services. The survey used a retrospective post-then-pre design to gather fathers’ perspectives on the Fatherhood is Sacred program and its influence on them. Among the men, more than 90% of them indicated that the program improved their self-confidence as a father, helped their parenting and relationship skills, supported them in their sobriety efforts (if this was an issue), and increased their connection to community services and groups of men who provided positive support (White and Brotherson, 2005). All of these items link well with the need to support and sustain healthy coparenting relationships in families.

Additionally, those surveyed also noted the program’s impact on their general wellbeing, with more than 80% of respondents noting lower levels of personal discouragement, improved anger management and an increased sense of belonging. For men with substance abuse concerns, over 70% of them indicated a reduction in varied substance use behaviors or relapse to past behaviors since the inception of the program. Program participants also reported improvements in employment circumstances, interactions with law enforcement, and feelings about their heritage. Importantly, 92% of the men indicated that their feelings about being a father improved, with more than 80% noting increased father involvement and support of their children. Also, they reported improvements in the quality of relationship with the child’s mother. Finally, the men surveyed also rated the Fatherhood is Sacred program as most useful among a variety of sources for individual guidance on parenting, and also rated it highest among 11 other organizational support sources for being useful to them in their parenting and support (White and Brotherson, 2005).

While more extensive and careful research is needed to better understand the Fatherhood is Sacred program, the initial findings from evaluative research efforts and investigations of programs with best practices suggest that the program has much promise for application and adaptation to other settings (White and Brotherson, 2005; Goodrow, 2015; Wilson et al., 2022).

## 4 Practical implications—best practice applications for Afro-Caribbean populations

The interesting question arising from this review is whether other populations beset by the vestiges of intergenerational and modern-day trauma, like some Afro-Caribbean families and communities, can benefit from this program or its related best practices. When considering the adaptation of fatherhood programs to the Afro-Caribbean context, there are several factors to bear in mind. Programs should be culturally and contextually relevant; address masculinity norms; utilize a strengths-based approach to fatherhood programming; allow for community engagement and collaboration; be holistic in their approach; adopt an intergenerational focus; and importantly, incorporate robust evaluation and impact measurement. For Afro-Caribbean men and families, there are a variety of obstacles to navigate and diverse family settings to consider when exploring opportunities for responsible fathering (Anderson and Daley, 2015). There is a substantive history of fatherhood programs supporting Black American fathers, and also some efforts for men in the Afro-Caribbean contexts (though these tend to be much more limited) (Lu et al., 2010; Karberg et al., 2017). Such programs in the Caribbean context include: Fatherhood Initiative by Rising Ground, which is a 3-month Program implemented in the Bronx, New York; Fathers Incorporated, which is Jamaican initiative that sought to promote positive fatherhood involvement in the 1990s but has since sought to focus on supporting disadvantaged children; the newly established Caribbean Fatherhood Coalition by Deeds Driven Dads in the Eastern Caribbean; and the “Affirming Fatherhood” Webinar Series organized by the Caribbean Male Action Network (CARIMAN) and Parenting Partners Caribbean (PPC).

One of the challenges with fatherhood related programs in the Caribbean and those implemented in Diaspora communities in the United States is that robust impact and evaluation measurement data is not readily available for most efforts (Karberg et al., 2017). Also, many of these programs are built on a deficits-based narrative and do not incorporate elements that resonate with the cultural traditions, values, and family structures prevalent in the Afro-Caribbean communities. While not a monolithic view, there is also a tendency among Afro Caribbean people, to feel greater cultural affinity and closeness to Africa compared to African Americans, who experienced more dilution of African roots over centuries in the U.S. (Thornton et al., 2017). This increases the challenge associated with adapting programs to an Afro-Caribbean context that were designed and implemented in the U.S. that target African Americans.

By and large, the Fatherhood is Sacred Program appears to be an example of a community-based program that fits with the key factors to consider already mentioned, with the final concern being that of cross-cultural adaptation. In the development field, there are multiple examples of evidence-based fatherhood and parenting programs that were developed in one cultural context and have been systematically adapted and implemented successfully in other cultural contexts. Accounting for differences in values, traditions, language, and barriers is fundamental. Examples of such programs include: the Healthy Dads, Healthy Kids (HDHK) Program, which was originally developed in Australia, and was culturally adapted as “Papás Saludables, Niños Saludables” for Hispanic families in the U.S.; the ParentCorps Program, which is an evidence-based parenting intervention originally designed for African American and Hispanic/Latino families living in low-income communities, which was culturally adapted for engagement with a wider cross-section of people; and Incredible Years Parenting Program, which was developed in the U.S., but has since been transported and culturally adapted for use in countries like Sweden, Norway, Netherlands, Portugal, and others (Booth and Lazear, 2015; Leijten et al., 2015; O’Connor et al., 2020).

Studies addressing cross-cultural adaptation with interventions to further culturally competent practice have pointed to the challenges, as well as the opportunities, related to cultural adaptation of behavioral interventions. Since culture occurs at multiple levels and is also fluid and ever-changing, the process of cultural adaptation becomes particularly complex and dynamic (Marsiglia and Booth, 2015). A culturally grounded approach can only be effective when it is centered around the lived experiences of the participants without compromising the effectiveness of the program. Both the models posited by the Southwest Interdisciplinary Research Center (SIRC) and the Centers for Disease Prevention and Control (CDC) postulate that adaptation in cross-cultural contexts is possible and can be effective (Marsiglia and Booth, 2015). As an example, in looking at cross-cultural similarities and differences in parenting, Lansford (2022) notes that sometimes entirely new programs are developed within a particular cultural context, but that it is more common that a program developed in one context is adapted for use in another context or population. Further, cultural adaptation of a program is deemed as necessary when seeking to apply it in a different setting, which fits squarely with the general premise of this paper.

While an in-depth discussion of this possibility is beyond the scope of this paper, it does suggest two important considerations for applying best practices from the Fatherhood is Sacred program in a Caribbean context. First, since the Fatherhood is Sacred program draws upon and reinforces cultural background and identity for Native populations in its application (Goodrow, 2015), any effort to implement the program in an Afro-Caribbean setting ought to review and incorporate relevant cultural background and identity factors for Caribbean families into the program like the tradition of storytelling, the vibrant music and other cultural factors. Second, usage of a cultural adaptation model (such as those outlined by SIRC or the CDC) to facilitate adjustment of program elements as needed to fit within an Afro-Caribbean context would be necessary and advance the program’s cultural relevance.

Another best practice lesson from the Fatherhood is Sacred program that also fits within an Afro-Caribbean context is the importance of acknowledging historical and intergenerational trauma with a focus on healing from such traumas and moving forward (Wilson et al., 2022). A key lesson learned from the Native American experience is that context matters. Native American and Afro-Caribbean populations share some similarities in historical experience. Both communities have experienced traumatic events that are pervasive and persistent, creating high levels of collective distress that have been passed through generations. For both populations, the resultant effects are evidenced in the displacement and damage caused to family systems, which provide the foundation of any society. Continuing patterns of absentee fatherhood and low father involvement have placed youths in these communities at risk for maladaptive behaviors.

The Fatherhood is Sacred program was developed to directly address the adverse effects of the abusive and culturally insensitive programs and policies directed at Native American peoples (Montgomery, 2015). It is important to treat intergenerational trauma, as with any trauma, directly, through identifying its effects on personal attitudes and family patterns, thereby facilitating parental awareness of lifespan implications and the collective effects of lost identity that can span generations (Wilson et al., 2022). In an Afro-Caribbean context, necessary adaptations would include an awareness of the legacies of enslavement, migration, racism and other policies that dramatically impacted today’s Caribbean populations (Morgan, 2016).

The Fatherhood is Sacred program and its expansion indicates that the development of a strong cultural model can be a critical ingredient to success. The program is grounded in traditional Native American values – this is a key factor in its primary appeal (Goodrow, 2015). In reviewing strategies for effective intervention approaches with Native populations, Wilson et al. (2022) emphasize the need to “include content that highlights and respects the cultures, heritages, beliefs, traditions, and histories of AIAN peoples” (p. 5). Restoring culture and identity as a Native American is one foundational principle of the program that in many ways accounts for its resonance and success among Native American populations (Montgomery, 2015; Sarche et al., 2020). Including the heritage and beliefs of Native American peoples through this program has provided fathers with a sense of identity (White and Brotherson, 2005).

Would it be possible to replace that particular program element of the cultural connections for Native Americans with that of Afro-Caribbean peoples? The culture of Afro-Caribbean people is a melting pot of varied and distant African traditions that were mixed with the European practices of slave masters, and more recently adapted based on the acculturation influences from North America (Bush, 2010). It would be likely impossible to identify in its purest form anything like the *Woope Sakowin* (Seven Laws), which are universal virtues of the Lakota Indian people and the foundation of Lakota culture (Brave Heart, 1999). In a study undertaken by Thornton et al. (2017), it was found that Black Caribbean people feel significantly closer to Black people in Africa than do African Americans. This cultural affinity may create an opportunity to superimpose some elements of African culture into this program. In the Caribbean, it is also possible to identify a unique set of Afro-Caribbean values—values that are rooted in a shared history of the people. Such values would include respect, especially for adults; non-entitlement; hard work; a drive to succeed; strong religious beliefs; and the acceptance of the extended family as a support system (Archibald, 2011). These are values upon which programs like the Fatherhood is Sacred program that encourage father involvement could be adapted to an Afro-Caribbean context. Archibald (2011) suggests that such “cultural tailoring” promotes program effectiveness, citing examples that this approach improves motivation to participate “when such interventions are culturally relevant and respectful of their culture” and also promotes “a sense of empowerment among parents” (p. 115).

Another best practice included in the Fatherhood is Sacred program includes incorporating a holistic, positive and strengths-focused perspective of fathers in families and communities. As noted by Montgomery (2015), the program explicitly seeks to engage men in understanding their roles are important, their leadership is needed, and they can bring solutions to families and communities. In doing so, the program fits well with the framework of generative fathering and the lifelong development of fathers. Hawkins and Dollahite (1997) note that scholars and practitioners, in evaluating models of fatherhood, typically focus attention on the deficiencies of fathers using a deficit-driven paradigm. As a result, such efforts are likely to find and focus on inadequate role performance by fathers. Cases of absentee fathers or fathers who fail to support families economically and emotionally are identified as being prevalent in the Afro-Caribbean culture (Boyne, 2005; Roopnarine, 2013; Jemison, 2015). In contrast, the generative fatherhood model incorporated in the Fatherhood is Sacred program focuses on seeing men as being willing and able to execute the work of responsible, healthy, and involved fathering, though often constrained by the context in which they must operate (including historic and contemporary traumatic events) (Hawkins and Dollahite, 1997).

An additional best practice that emerges from the Fatherhood is Sacred program is its intentional focus on the value and importance of fathers for child and family well-being as a priority versus other topics (Wilson et al., 2022). While other elements are included in the program, this focus on men and their sacred roles as fathers and their contributions to children is highly emphasized (Goodrow, 2015). This emphasis aligns well with a key practice identified in Wilson et al.’s (2022) review of programming for Native populations, which notes that “fathers primarily participate in fatherhood programming in hopes of promoting the best possible outcomes for their children” (p. 6). The Fatherhood is Sacred program seeks to restore confidence in fathers’ role and contributions that has been lost or diminished, and heralds the accomplishments of Native American men, the *Akicitas* (in Lakota culture), who were also caregivers (Montgomery, 2015). Daly (1996) presents research findings that illustrate that most fathers have strong feelings for their children, believe that their families are more important than their work and want to spend more time with and caring for their children. In Afro-Caribbean family systems where the efforts of mothers are often most emphasized, adopting such an intentional focus on fathering would likely be important to appeal to men and their interests.

Yet another best practice in the Fatherhood is Sacred program that has relevance includes its explicit focus on identifying and cultivating men’s potential and their strengths in family life. Too often, populations that have experienced trauma become characterized primarily by narratives of loss, dysfunction or difficulty. However, Wilson et al. (2022) note, “Experts and previous research support the idea of using strengths-based practices and incorporating traditional AIAN values like reciprocity, respect, kindness, and fairness” (p. 6). While there have been few dedicated studies in recent years on the issue of fathering in the Afro-Caribbean community, the existing work has focused attention on the deficit-driven model of fatherhood, drawing attention almost solely to the prevalence of fatherlessness in the region and the adverse impact this has had on developmental outcomes for children and society (Roopnarine, 2013; Jemison, 2015). Few studies have approached the challenge from a different angle, a strengths-based approach, that evaluates the willingness and capacity of Afro-Caribbean fathers to be involved parents and coparents, while also highlighting the historical, cultural, and social factors that may discourage men in their family efforts.

## 5 Conclusion

In summary therefore, the specific aspects of the Fatherhood is Sacred program that could potentially be adapted to make it more culturally relevant for Afro-Caribbean communities could include the incorporation of Afro-Caribbean cultural teachings, practices and activities; addressing the unique historical experiences and contemporary challenges facing Afro-Caribbean fathers and families, such as the traumatic legacies of slavery, migration and racism; engaging Afro-Caribbean community leaders, and fathers, with a view to identifying cultural mismatches and tailoring program content, metaphors, and delivery methods appropriately; and finally, the examination of family and gender norms to enhance relevance, while considering urban/rural and island contexts.

We would suggest that these and other best practices provide a direction for adapting a program like Fatherhood is Sacred for use in Afro-Caribbean contexts. To strengthen the contributions of men in Caribbean families and communities, light needs to be focused on examples of generative fatherhood in this society, notwithstanding the challenges, with a view to increasing understanding of how such behavior can be supported and replicated. Built on the foundation of empowering men to see themselves as good fathers and move toward that vision, the Fatherhood is Sacred program teaches men how to support the next generation of fathers and views this forward-looking strategy as being integral to the future growth and development of children. The Afro-Caribbean community can, without a doubt, benefit from this visioning exercise and the possibility of similar approaches. Naturally, when contemplating the use of instruments to measure concepts across different groups, consideration must be given to concepts of configural invariance, metric invariance, and scalar equivalence.

There are still many unanswered questions about the effects of intergenerational trauma on family systems and coparenting. Continued research on Native American families and communities is providing much needed answers to some of these questions. These answers can benefit other communities affected by historical trauma, such as Afro-Caribbean populations. Culturally informed approaches to treat family and community effects of such trauma need to be identified and promoted. We suggest there are meaningful possibilities to adapt interventions like the Fatherhood is Sacred program and utilize its best practices to address the needs of populations experiencing similar coparenting and familial challenges, such as the Afro-Caribbean community. In doing so, we can respond in a meaningful way to those whose cry for help spans generations.

## Data availability statement

The original contributions presented in the study are included in the article/supplementary material, further inquiries can be directed to the corresponding author.

## Ethics statement

The studies involving humans were approved by Institutional Review Board, North Dakota State University. The studies were conducted in accordance with the local legislation and institutional requirements. The participants provided their written informed consent to participate in this study.

## Author contributions

SH: Conceptualization, Writing – original draft. SB: Conceptualization, Supervision, Validation, Writing – review & editing.
